# Aryl-1,3,5-triazine ligands of histamine H_4_ receptor attenuate inflammatory and nociceptive response to carrageen, zymosan and lipopolysaccharide

**DOI:** 10.1007/s00011-016-0997-z

**Published:** 2016-10-20

**Authors:** Szczepan Mogilski, Monika Kubacka, Dorota Łażewska, Małgorzata Więcek, Monika Głuch-Lutwin, Małgorzata Tyszka-Czochara, Karolina Bukowska-Strakova, Barbara Filipek, Katarzyna Kieć-Kononowicz

**Affiliations:** 1 Departament of Pharmacodynamics, Jagiellonian University Medical College, Medyczna 9, 30-688 Kraków, Poland; 2 Department of Technology and Biotechnology of Drugs, Jagiellonian University Medical College, Medyczna 9, 30-688 Kraków, Poland; 3 Department of Pharmacobiology, Faculty of Pharmacy, Jagiellonian University Medical College, Medyczna 9, 30-688 Krakow, Poland; 4 Department of Radioligands, Faculty of Pharmacy, Jagiellonian University Medical College, Medyczna 9, 30-688 Kraków, Poland; 5 Department of Medical Biotechnology, Faculty of Biochemistry, Biophysics and Biotechnology, Jagiellonian University, Krakow, Poland; 6 Department of Clinical Immunology and Transplantology, Polish-American Institute of Pediatrics, Medical College, Jagiellonian University, Krakow, Poland

**Keywords:** Histamine, H_4_ receptor, Inflammation, Pain, Edema, Cytokine, Triazine

## Abstract

**Objective and design:**

Histamine H_4_ receptor (H_4_R) offers a great potential for new therapeutic strategies for the treatment of inflammation-based diseases. The aim of this study is to present the pharmacological profile of two recently synthesized ligands of H_4_R with particular reference to their anti-inflammatory and analgesic activity.

**Materials and subjects:**

We used mice and rats in the in vivo tests. We also used murine RAW 264.7 cells and isolated guinea-pig ileum in in vitro test.

**Treatments:**

In the in vivo tests, animals were pre-treated with the increasing doses of investigated compounds (12.5, 25 and 50 mg/kg) and reference compounds: JNJ7777120 (25 mg/kg), indomethacin (10 mg/kg). Macrophages were pre-treated with two concentrations of tested compounds 100 and 10 µM.

**Methods:**

We examined anti-inflammatory and analgesic effects of the new H_4_R antagonists in the in vivo models of inflammation induced by carrageenan or zymosan. We assessed the level of cAMP and release of cytokines, ROS and NO in lipopolysaccharide (LPS)-stimulated RAW 264.7 macrophages. Moreover, we assessed the affinity of the investigated compounds for histamine H_1_ receptor in functional studies.

**Results:**

Both investigated compounds reduced paw edema, mechanical and thermal hyperalgesia in the carrageenan-induced acute inflammation. Moreover, administration of the investigated compounds resulted in decreased granulocyte influx and attenuated nociceptive reaction in the zymosan-induced peritonitis model. In the same model of inflammation, the investigated compounds reduced vascular permeability; however, this effect was observed only after the highest applied dose. Furthermore, the test compounds had no impact on cell viability in the experiments on RAW 264.7 macrophages. In these cells, stimulated with LPS, the test compounds decreased reactive oxygen species (ROS) production. They increased the cellular concentration of cAMP and attenuated the production of inflammatory cytokines such as TNFα and IL-1β. All results were comparable to those obtained for the reference compound JNJ7777120 with the exception of the impact on NO production. Nevertheless, this effect was similar to that obtained for the other reference compound rolipram, which is a phosphodiesterase 4 (PDE 4) inhibitor. Further experiments revealed that both of the investigated compounds possessed relatively low affinity for histamine H_1_ receptor and do not inhibit the activity of the PDE 4B1 enzyme. In addition, all the effects of the investigated compounds in in vivo experiments were observed at doses that did not cause neurologic deficits in rotarod test and did not reduce spontaneous locomotor activity.

**Conclusions:**

Our results demonstrate the anti-inflammatory and analgesic activity of the new aryl-1,3,5-triazine derivatives, which are primarily H_4_R–dependent.

## Introduction

The histamine H_4_ receptor (H_4_R) is one of the four known subtypes of histamine receptors (H_1_R–H_4_R). This receptor is a member of class A G-protein coupled receptors (GPCRs). On the molecular level the activation of H_4_R by histamine results in the liberation of Gα_i/o_ subunits, subsequent inhibition of adenylyl cyclase activity, and decreased concentration of cAMP. The downstream signals of this pathway involve changed activity of protein kinase A and transcription of genes regulated by cAMP-responsive elements [1, 2]. Strong increase in the phosphorylation of mitogen-activated protein kinase associated with H_4_R stimulation was also reported [3]. Moreover, the Gβγ subunit of G-protein induces calcium^2+^ mobilization through phospholipase C activation [4]. An increased concentration of calcium^2+^ leads to actin polymerization enabling the cells with an expression of H_4_R to change the cell shape and to migrate into the site of inflammation [5]. Research into GPCRs reveal that these receptors can exist in multiple active receptor conformations, which are associated with different signaling pathways and couple to multiple downstream effector proteins [6, 7]. Regarding H_4_R, β-arrestin2 recruitment that occurs independently of G protein was shown [8]. Furthermore, it was presented that certain ligands are able to preferentially activate one pathway with the opposite effect or no impact on the other-biased H_4_R ligands [8, 9]. There is also evidence of constitutive activity of H_4_Rs, which means that these receptors are able to undergo agonist-independent isomerization from an inactive state to an active one [3, 10]. Thereby, the biological response in the absence of a bound ligand can be inhibited by H_4_R inverse agonists—the ligands that stabilize the inactive receptor conformation and are therefore able to reduce constitutive activity. It is important that primarily human H_4_R receptor possesses a high constitutive activity, whereas canine, murine and rat H_4_R orthologs show substantially lower activity in the absence of an agonist [11, 12].

The H_4_Rs are predominantly expressed in a variety of immune cells. The majority of them such as dendritic cells, neutrophils, mast cells, eosinophils, basophils, monocytes and CD4^+^ T cells are derived from the hematopoietic stem cells, which also present expression of this receptor [13, 14]. Furthermore, the H_4_Rs were detected in the enteric and central nervous system as well as on dermal fibroblasts and nerves of the human nasal mucosa [1]. The H_4_R is involved in many functional inflammatory responses mediated by histamine, including chemotaxis, cell recruitment, increased expression of adhesion molecules and modulation of cytokine and chemokine release [2, 15, 16].

Research into the first potent and selective H_4_R antagonist (JNJ 7777120) provided a large body of evidence that inhibition of H_4_R function (antagonists, inverse agonists) could result in attenuating an inflammatory response. This compound, along with other H_4_R antagonists, has shown activity in models of asthma, dermatitis, pain, and pruritus among others [17–20]. It supports the hypothesis that the histamine H_4_R offers a great potential for new therapeutic strategies for the treatment of inflammation-based diseases.

The aim of this report is to present the pharmacological profile of two recently synthesized ligands of H_4_R with particular reference to their anti-inflammatory and analgesic activity. These compounds (4-(4-Methylpiperazin-1-yl)-6-(4-chloro-phenyl)-1,3,5-triazin-2-amine as 1 and 4-(4-Methylpiperazin-1-yl)-6-(4-bromo-phenyl)-1,3,5-triazin-2-amine as 2) have been chosen from the series of previously described 1,3,5-triazine derivatives. In the previous experiments both compounds showed submicromolar affinities for the H_4_R and caused a blockade of the histamine-induced cAMP reduction in CHO-h H_4_R-cAMPzen cells co-treated with forskolin. Moreover, tests for their interaction with H_3_Rs, which show the highest sequence homology to the H_4_R, revealed low affinity for these receptors. Such results entitled to classify the compounds as selective H_4_R antagonists [21].

## Materials and methods

### Animals

The experiments were carried out on adult male Albino Swiss mice (CD-1, 18–25 g), male Wistar rats [Krf: (WI) (WU), 180–250 g], and male guinea pigs (Outbred CV, 300–400 g). The animals were housed in plastic cages in a room at a constant temperature of 20 ± 2 °C, under light/dark (12:12) cycle and had free access to standard pellet diet and water. The experimental groups consisted of 6–12 animals; all the animals were used only once and they were killed by cervical dislocation immediately after the assay. The rats and the guinea pigs were previously anesthetized with sodium pentobarbital (60 and 37 mg/kg, respectively). The minimum number of animals needed to obtain definite and normally distributed results was used. Behavioral measures were scored by trained observers, which were blind to experimental conditions. The treatment of laboratory animals in the present study was in full accordance with the respective Polish regulations. All procedures were conducted according to guidelines of ICLAS (International Council on Laboratory Animal Science) and approved by the Local Ethics Committee of the Jagiellonian University in Cracow (ZI/121/2011).

### Drugs and chemicals

**Fig. 1:**
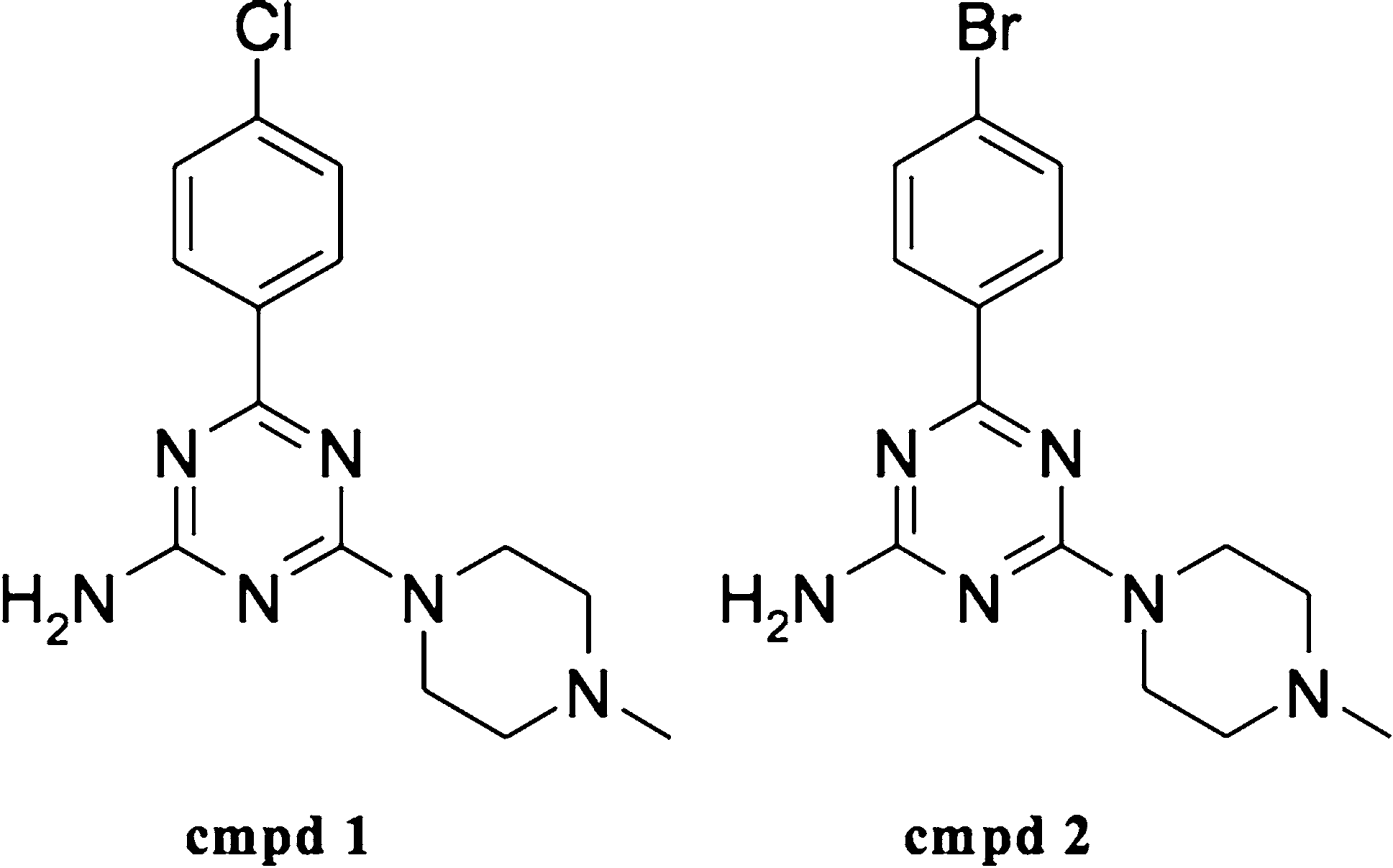
Schematic structure of the tested aryl-1,3,5-triazine derivatives

Two compounds, aryl-1,3,5-triazine derivatives: 1 and 2 (Fig. 1) and JNJ7777120 were synthesized in the Department of Technology and Biotechnology of Drugs, Jagiellonian University Medical College. Synthesis of the investigated compounds was described previously [21]. For behavioral experiments, investigated compounds and reference compounds were suspended in a 1 % aqueous solution of Tween 80 and administered by the intraperitoneal (i.p.) route 30 min before the test. In the zymosan-induced peritonitis, the reference and the investigated compounds or vehicle (DMSO/PBS, 1:3) were administrated subcutaneously (s.c.) 30 min prior to zymosan. Control animals (negative control) were given an appropriate amount of vehicle (1 % aqueous solution of Tween 80; i.p.) 30 min before the test. Injections were made in a volume of 10 ml/kg (mice) or 2 ml/kg (rats).

The following drugs and reagents were used: carrageenan (Viscarin, FMC BioPolymer, USA), DMSO (Methyl sulfoxide, P.O.Ch., Poland), Evans blue (Sigma-Aldrich, Germany), histamine hydrochloride (Sigma-Aldrich, Germany), indomethacin (Sigma-Aldrich, Germany), LPS (Lipopolysaccharides from *Escherichia coli*, Sigma-Aldrich, Germany), PBS (Perkin Elmer, USA), Rolipram (Sigma-Aldrich, Germany), Trypan blue (Sigma-Aldrich, Germany), zymosan (Sigma-Aldrich, Germany).

Other chemicals used (e.g., in Krebs solution) were obtained from POCH (Polish Chemical Reagents, Poland) or were included in the commercially available kits presented in the description of the particular method.

### Protocols for in vivo experiments

#### Carrageenan-induced paw oedema, mechanical and thermal hypernociception

The acute, local inflammation and paw oedema was induced by subplantar injection of 0.1 ml of 1 % carrageenan (made in PBS) into the rat right hind paw. The paw volume was measured by the dislocation of the water column of the plethysmometer (Plethysmometer 7140, Ugo Basile) before (V_0_) and at 1, 2 and 3 h after subplantar injection of carrageenan (V_1_, V_2_, V_3_). The increase in volume of the inflamed paw was determined by subtracting the volume measured before carrageenan (V_0_) from the observed value at 1, 2 and 3 h (V_1–3_) and expressed as a percentage: % oedema = (V_(1–3)_–V_0_) × 100/V_0_.

Additionally, the pain pressure threshold was used to measure the hyperalgesic response to mechanical stimuli. Increasing pressure was applied to the dorsal surface of the right hind paw by an automated gauge (Analgesy Meter 37215, Ugo Basile) according to the method of Randall and Selitto. The intensity of the applied force, in grams, was recorded when the paw was withdrawn (withdrawal threshold). For each animal paw withdrawal thresholds were recorded before carrageenan administration and 3 h afterwards [22]. The results were expressed as % of initial reaction, with nociceptive reaction before carrageenan administration considered as 100 % response.

The hyperalgesic response to thermal stimuli was determined by using a plantar test apparatus (Commat Ltd., Turkey). Rats were placed individually in plexiglas chambers and allowed to acclimatize for 20–30 min before testing. The radiant heat was positioned under the chamber floor directly beneath the hind paw and the latency to paw withdrawal was automatically recorded by a photocell and an electronic timer. The intensity of the radiant heat was adjusted to achieve baseline latencies of 10–15 s and a cut-off time of 30 s was preset in order to avoid tissue damage. Unresponsive animals after 30 s (cut-off time) were discarded. Three subsequent applications of heating stimulus were done, separated by 1- to 2-min intervals, and the mean of these measures was taken. The paw withdrawal latency was recorded for each animal before carrageenan administration and 1, 2 and 3 h afterwards [19].

Rats were pre-treated with the dose–response of investigated compounds (12.5, 25 and 50 mg/kg and reference compounds: JNJ7777120 (25 mg/kg), indomethacin (10 mg/kg).

#### Zymosan-induced peritonitis

The acute, murine peritonitis was induced by an intraperitoneal (i.p.) injection of freshly prepared zymosan A (40 mg/kg, Sigma-Aldrich). The reference and investigated compounds or vehicle (DMSO/PBS, 1:3) were administrated s.c. 30 min prior to irritant. Immediately after the zymosan injection each mouse was placed in a separate cage and pain symptoms were tested by counting characteristic body writhes. Numbers of body writhes were scored for 45 min. After observations the animals were returned to their original cages. Four hours after zymosan injection, animals were euthanized, the peritoneal cavities were washed with 1.5 ml of ice-cold PBS (pH 7.2) and the lavage was collected in a heparin-containing tube [23]. The number of migrated leukocytes was determined with Countess Automated Cell Counter (Invitrogen), by taking an aliquot (10 µl) of the lavage fluid. The sample was mixed with 10 μl of trypan blue, and pipetted into Countess chamber slide. The results were expressed as total cell concentration per 1 mL. The samples that were visibly red were not included in the analysis.

Some cell populations in the lavage fluid were determined by the method of flow cytometry. The cell suspensions (1 ml) were filtered with 70 μm strainer, depleted of erythrocytes by use of a hypotonic solution, centrifuged (600 g, 10 min, 4 °C), resuspended in PBS (Lonza) with 2 % FBS, and stained for 20 min on ice. Following antibodies were used for cell immunophenotyped with following combination of mAbs: (1) NK1.1 FITC; Ly6G FITC; CD43 PE; CD11b-PE-CF954 (clone M1/70, BD Biosciences); Ly6C PerCP-Cy5.5; MHC-II PE-Cy7; F4/80 APC; CD11c AlexaFluor 700; CD14 APC-Cy7; Hoechst 33342; CD45 pacific Orange; (2) NK1.1 FITC; CD25 PE; CD4 PE-CF945; CD3 PE-Cy7; CD8 APC-Cy7; CD45R-V450 (clone RA3-6B2, BD Biosciences); CD45 Pacific Orange;The stained cells were analyzed using LSRII flow cytometer (BD Biosciences), with FACSDiva (BD Biosciences) and Diva software.

Cell populations were defined based on:

- granulocytes: CD11b +/Ly6G +/SSChigh
- monocytes: CD11b+/Ly6G-NK1.1-SSClow/Ly6Cdim/+/CD43±
  - classical monocytes: CD11b+/Ly6G-NK1.1-SSClow/Ly6Chigh/CD43-/CD14++/MHC-II-
  - intermediate monocytes: CD11b+/Ly6G-NK1.1-SSClow/Ly6Cmed/CD43med/CD14+/MHC-II-
  - non classical monocytes: CD11b+/Ly6G-NK1.1-SSClow/Ly6Cdim/CD43+/CD14-/MHC-II+
- plasmacytoid DC: CD11b+/Ly6G-NK1.1-SSChigh/CD14-MHC-II+/CD11c+
- myeloid DC: CD11b+/Ly6G-NK1.1-SSChigh/CD14+/MHC-II+/CD11c+
- macrophages: CD11b+/Ly6G-NK1.1-SSChigh/CD14++/MHC-IImed/F4/80++

Evans blue, suspended in saline (10 mg/ml), was injected (i.v.) into caudal vain of another group of mice (0.2 ml/mouse) to evaluate the influence of investigated compounds on vascular permeability in zymosan-induced inflammation. Evans blue injection was immediately followed by administration of zymosan A as described above. Thirty minutes later, the animals were killed and their peritoneal cavities were lavaged with 1.5 ml of saline. The lavage fluid was centrifuged and the absorbance of dye in the supernatant was measured at 620 nm with a Multiscan Go (Thermo Scientific).

Investigated compounds and reference compounds were administrated at the same doses as in method 2.3.1 (Carrageenan-induced inflammation).

#### Influence on spontaneous locomotor activity

The locomotor activity test was performed using activity cages (40 × 40 × 31 cm) supplied with I.R. horizontal beam emitters (Activity Cage 7441, Ugo Basile, Italy) connected to a counter for the recording of light-beam interrupts. 30 min before the experiment the mice were pretreated (i.p.) with the test compound at a dose of 50 mg/kg (the highest dose used in the previous tests for analgesic and anti-inflammatory activity), and were then individually placed in the activity cages. The number of light-beam crossings was counted in each group during the next 30 min in 10-min intervals [24].

#### Influence on motor coordination in the rotarod test

The test was performed according to the method previously described [24]. The test was carried out on the semi-automatic rotarod apparatus (Rotarod apparatus Panlab/Harvard Apparatus, LE 8200, Spain). The mice were trained for 3 days on the rod rotating at a constant speed of 18 rotations per minute (rpm). During each training session, the animals were placed on the rod for 3 min with an unlimited number of trials. On the test day (24 h after the final training trial), 30 min before the rotarod test the mice were pretreated (i.p.) with the test compound (50 mg/kg). Then the animals were tested on the rotarod revolving at 6, 18 and then 24 rpm. Motor impairments, defined as the inability to remain on the rotating rod for 1 min were measured at each speed.

### Protocols for in vitro experiments

#### Influence on LPS-stimulated RAW 264.7 cells

The RAW 264.7 mouse macrophage cell line was obtained from ATCC and maintained according to supplier documentation. The RAW 264.7, mouse macrophage cell line, were grown in culture medium Dulbecco’s Modified Eagle’s Medium (DMEM) (ATCC 30-2002) supplemented with 10 % fetal bovine serum (FBS) (Thermo HyClone SV30160.03), 100 IU/ml penicillin, 100 µg/ml streptomycin (Life Technologies 15140122). The cells were cultured in 5 % CO_2_/95 % air, 100 % humidity, at 37 °C until the adhered monolayer reached 70 % confluency (usually 1–2 days). The monolayer was once washed with calcium/magnesium-free phosphate buffered saline (Ca^++^/Mg^++^-free PBS, Sigma D8537). The cells were dislodged from the flasks with the scraper and then poured into 10 mL of culture medium. Cells were counted with a Countess^®^ Automated Cell Counter (Life Technologies) and diluted to the required density. Viability was determined with trypan blue and routinely found to be near 100 %. The cells 1 × 10^5^ were seeded into 96-well plates (Falcon 353872), and the cells 4 × 10^5^ were seeded into 24-well plates (Greiner bio-one 662160) in incubation medium Dulbecco’s Modified Eagle’s Medium (DMEM, ATCC 30-2002) supplemented with 10 % heat inactivated fetal bovine serum FBS (FBS, Sigma F4135) with 100 IU/ml penicillin and 100 µg/ml streptomycin (Life Technologies 15140122). After initial seeding, the cells were allowed to adhere to the bottom of the wells by further culture for 24 h at 37 °C in 5 % CO_2_/95 % air.

All tested compounds were dissolved in dimethyl sulfoxide (DMSO) with stock concentrations of 10 mM. The compounds were incubated for 15 min in an ultrasound water bath. Tenfold dilutions were prepared in phosphate buffered saline from the stock solution (PBS, Sigma). Macrophages were pre-treated with two concentrations of tested compounds 100 and 10 µM and the final concentration of DMSO was respectively 1 and 0.1 %. JNJ7777120 was used at the same concentrations as the studied compounds. Two hours afterwards the 5 µg/ml lipopolysaccharide (LPS) from Gram-negative bacteria *Escherichia coli* serotype 0111:B4 (Sigma L4391) was added to cells and incubation was continued for 24 h. All compounds were filtered 0.2 µm (Sarstedt 83.1826.001). Negative controls were cells with no added LPS. All experiments were performed in triplicates, in two independent assays [25].

##### Cytotoxicity assay

The bioluminescent ToxiLight bioassay (Lonza) is a cytotoxicity highly sensitive assay designed to measure cell membrane damage. After 24 h of treatments, 5 µl of the clear fluid above a sediment was deposited in a 384-well plate (Perkin Elmer). Then 20 μl of the Adenylate Kinase Detection Reagent (AKDR) were added to each well and the plates were shaken. As a positive control for lysis 10 % Triton X-100 (Sigma-Aldrich) in medium was used, the negative control was medium alone. After 5-min incubation of the supernatant with the AKDR, the luminescence was measured in a plate reader (POLARstar Omega, BMG Labtech). The results were expressed as percent of positive control, which corresponds to the percentage of dead cells with respect to the control sample.

##### Nitrite assay

Nitrites were measured in culture medium supernatants after 24 h of incubation with the compounds. The fluorometric assay of nitrite is based on the reaction of nitrite with 2,3-diaminonaphthalene (DAN) to form fluorescent 2,3-naphthotriazole. A 200 µM working nitrite standard was prepared from a 2.0 mM sodium nitrite stock solution in endotoxin-free deionized water (Sigma-Aldrich). A working DAN solution of 50 µg ml^−1^ was prepared by diluting a 20 mg ml^−1^ stock solution with 0.62 M HCl. All assays were conducted in 96-well black plates (Perkin Elmer). In each well, 20 µl of standard or 30 µl sample, were added, respectively, to 80 or 70 µl of endotoxin-free deionized water. Then, 10 µl of working DAN solution was added to each well and the plates were shaken. The plates were incubated at 23 °C for 10 min. After then, 20 µl of 2.8 M NaOH was added to each well, and the plates were shaken again. Then the plate was incubated in the dark for 1 min and measured in a fluorescence plate reader (POLARstar Omega, BMG Labtech) with an excitation of 355 nm and an emission of 460 nm. The results were expressed as percent of control sample (the solvent).

##### Reactive oxygen species assay

Intracellular reactive oxygen species (ROS) were measured using the 6-carboxy-2,7-dichlorodihydrofluorescein diacetate (DCFH-DA) (Sigma-Aldrich). After 24 h of incubation with the compounds, the cells were washed three times by phosphate buffered saline (PBS, Sigma-Aldrich). Then, 100 µl of 10 µM DCFH-DA solution in PBS was added to each well and the plates were shaken. The plates were incubated at 37 °C for 30 min. Fluorescence intensity was measured with excitation wavelength of 485 nm and emission wavelength of 520 nm using a plate reader (POLARstar Omega, BMG Labtech). The results were expressed as percent of control sample (the solvent).

##### Cytokine and cAMP assay

The concentrations of TNFα and IL-1β in the medium after incubation with tested compounds were determined using ELISA kits, according to the manufacturer’s instructions (Cell Sciences and RayBio). The intracellular level of cAMP was determined using the EIA Direct cyclic AMP kit, a competitive immunoassay for the quantitative determination of cyclic AMP in samples treated with 0.1 M HCl (Sigma-Aldrich).

#### Histamine H_1_ receptor functional assay

An 15-cm ileum segment was excised from the small intestine of male guinea pigs and immersed into a Krebs solution (NaCl 120 mM, KCl 5.6 mM, MgCl_2_ 2.2 mM, CaCl_2_ 2.4 mM, NaHCO_3_ 19 mM, glucose 10 mM). After the first 5-cm length closest to the ileo–caecal junction had been discarded, 2-cm-long fragments were cut. Each segment of the ileum was placed in a 20-ml chamber of tissue organ bath system (Tissue Organ Bath System–750 TOBS, DMT, Denmark) filled with the Krebs solution at 37 °C, pH 7.4, with constant oxygenation (O_2_/CO_2_, 19:1), stretched by means of closing clips between the metal rod and the force–displacement transducer. The preparations were allowed to stabilize in organ baths for 60 min under a resting tension of 0.5 g, washing every 15 min with fresh Krebs solution. After the equilibration period a cumulative concentration-response curve was constructed for histamine (10 Nm–10 μM) [26]. Following the first curve, tissues were incubated with one of the concentrations of tested compounds for 15 min and the next cumulative concentration curve to the agonist was obtained. Only one concentration of the potential antagonist was tested in each piece of tissue. The experiment was repeated four to eight times [27].

#### The phosphodiesterase 4B1 (PDE 4B1) activity assay

Inhibition of PDE4B1 was measured using PDElight HTS cAMP phosphodiesterase assay kit (PDELightTM, Lonza) according to manufacturer’s recommendations. cAMP measurements were performed with homogeneous TR-FRET immunoassay using the LANCE Ultra cAMP kit (PerkinElmer, USA). Luminescence was measured in a multifunctional microplate reader (POLARstar Omega, BMG Labtech, Germany). Test and reference compounds were dissolved in dimethyl sulfoxide (DMSO) at a concentration of 1 mM and further diluted in assay buffer (10 mM Tris–HCl, 10 mM magnesium chloride and 0.05 % Tween-20; pH 7.4). 10U PDE 4B1 (BPS Biosciences) in appropriate buffer was incubated with reference and tested compound for 20 min. After incubation, the cAMP substrate (final concentration 1.25 µM) was added and incubated for 1 h. Then PDELight AMP Detection Reagent was added and incubated 10 min. All reactions were carried out at 37 °C in white-walled, 96 half area-well plates which were obtained from Perkin Elmer.

Rolipram and Irsogladine were used as standards for inhibition potency. Compounds and standards were tested in screening assays in two concentrations 50 and 100 μM. The percentage of inhibition was calculated using DMSO as vehicle control.

### Data analysis

Data are presented as mean ± standard error of the mean (SEM). The vast majority of data was analyzed using GraphPad Prism Software (v.5). Statistically significant differences between groups were calculated using one-way analysis of variance (ANOVA) and the post hoc Dunnett’s multiple comparison test or two-way analysis of variance (ANOVA) and the post hoc Bonferroni’s comparison when appropriate. The Shapiro–Wilk test was used to check sample’s normality. The criterion for significance was set at *p* < 0.05. The log-probit method was applied to statistically determine the ED_50_ values, which are accompanied by their respective 95 % confidence limits [24].

In the experiments on isolated tissues, contractile responses to the stimulation by the agonists (in the presence or absence of tested compounds) are expressed as a percentage of maximal histamine effect (*E*_max_ = 100 %), reached in the concentration-response curves obtained before incubation with the tested compounds. Data are the mean ± SEM of at least three separate experiments. Schild analysis was performed, and when the slope was not significantly different from unity, the pA_2_ value was determined [22].

## Results

### Carrageenan-induced paw oedema, mechanical and thermal hypernociception

Subplantar injection of carrageenan induced an edema observed as an increased volume of the injected hind paw, which peaked 3 h after the injection. At this time edema increased by approximately two times in the inflamed paw (1.15 ± 0.05 vs 2.27 ± 0.06 cm^3^; 97.39 % increase in paw volume). Whereas, the increase of paw volume 1 and 2 h after carrageenan injection reached 41.74 and 75.65 %, respectively.

**Fig. 2:**
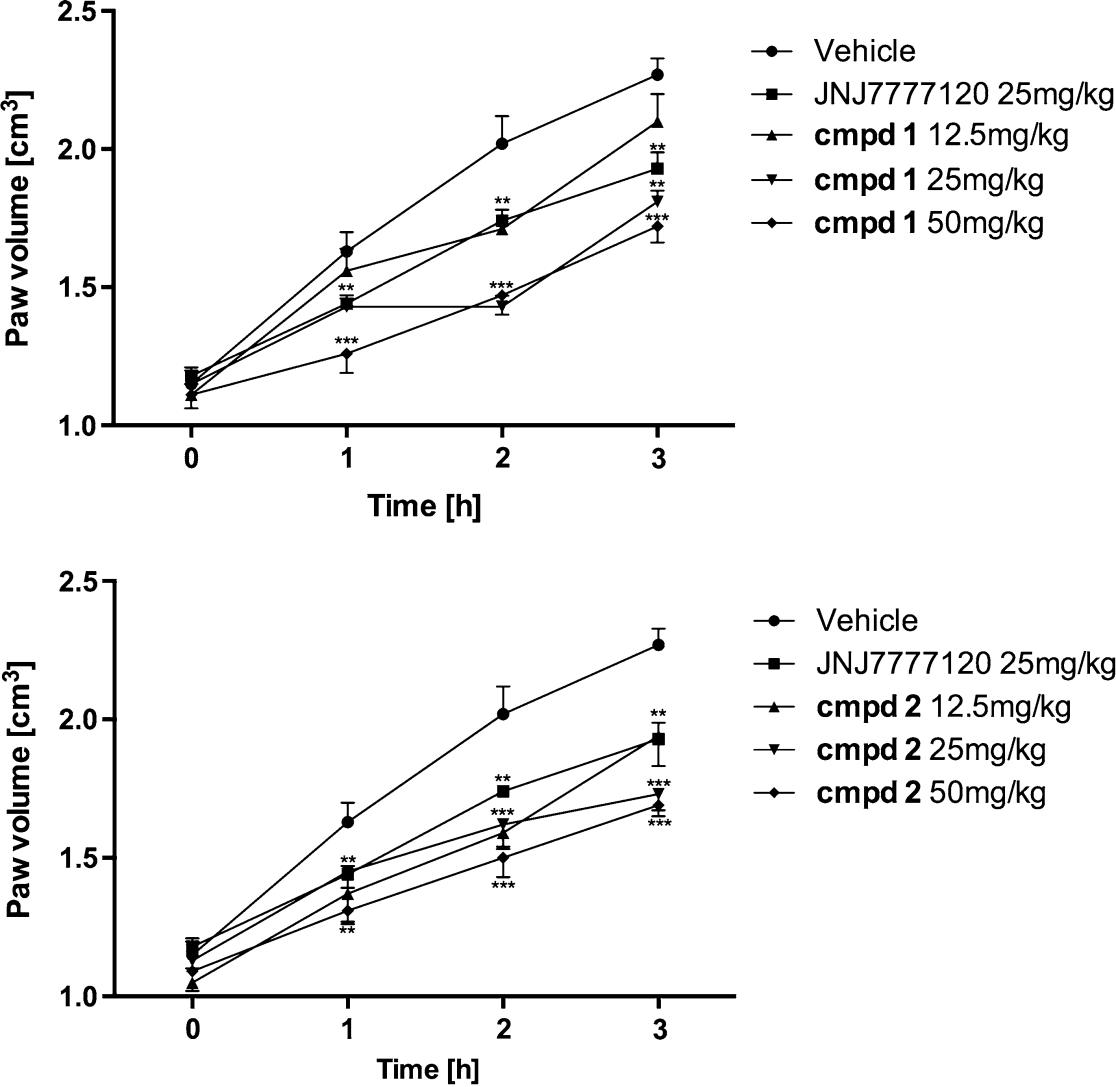
Effect of the tested aryl-1,3,5-triazines and reference compound JNJ7777120 on carrageenan-induced oedema. Data are expressed as mean ± S.E.M. for eight animals. Statistical analysis: two-way ANOVA followed by post hoc Bonferroni test. Statistical significance (*p <  0.05, **p <  0.01, ***p <  0.001)

Figure 2 shows the time course of rat paw edema development after the prior administration of compound 1, compound 2 or JNJ7777120 (25 mg/kg). All these agents were able to reduce the paw edema at all measured time points compared to the vehicle. The pretreatment with compound 1 at a dosage of 12.5 mg/kg reduced the paw edema by 2.94, 28.55 and 8.41 %, 1, 2 and 3 h following carrageenan injection, respectively. The effect of the higher dose of 25 mg/kg was more significant (41.66, 67.81 and 41.07 % of edema reduction); however, the highest dose of 50 mg/kg was not associated with an excessive increase of edema reduction compared to the dose of 25 mg/kg and the obtained values of edema reduction were 67.63, 57.13 and 43.57 %. The pretreatment with compound 2 resulted in effects similar to those observed for compound 1. The dose of 12.5 mg/kg reduced the edema formation by 27.00, 32.03 and 12.96 %, the dose of 25 mg/kg reduced the edema by 32.17, 42.68 and 45.48 %, while the dose of 50 mg/kg reduced the edema by 51.65, 50.28 and 43.48 % at 1-, 2- and 3-h time points, respectively. The effect of JNJ7777123 was included in the same range of activity as that observed for investigated compounds. However, JNJ7777123 was more active 3 h after carrageenan injection (63.55 % edema inhibition). The inhibition of edema formation 1 and 2 h after carrageenan injection reached 22.03 and 47.45 %. The most pronounced inhibition of paw edema was observed for indomethacin (59.46, 75.94 and 75.23 % of edema inhibition at 1, 2 and 3 h time points of the development of inflammation).

**Fig. 3:**
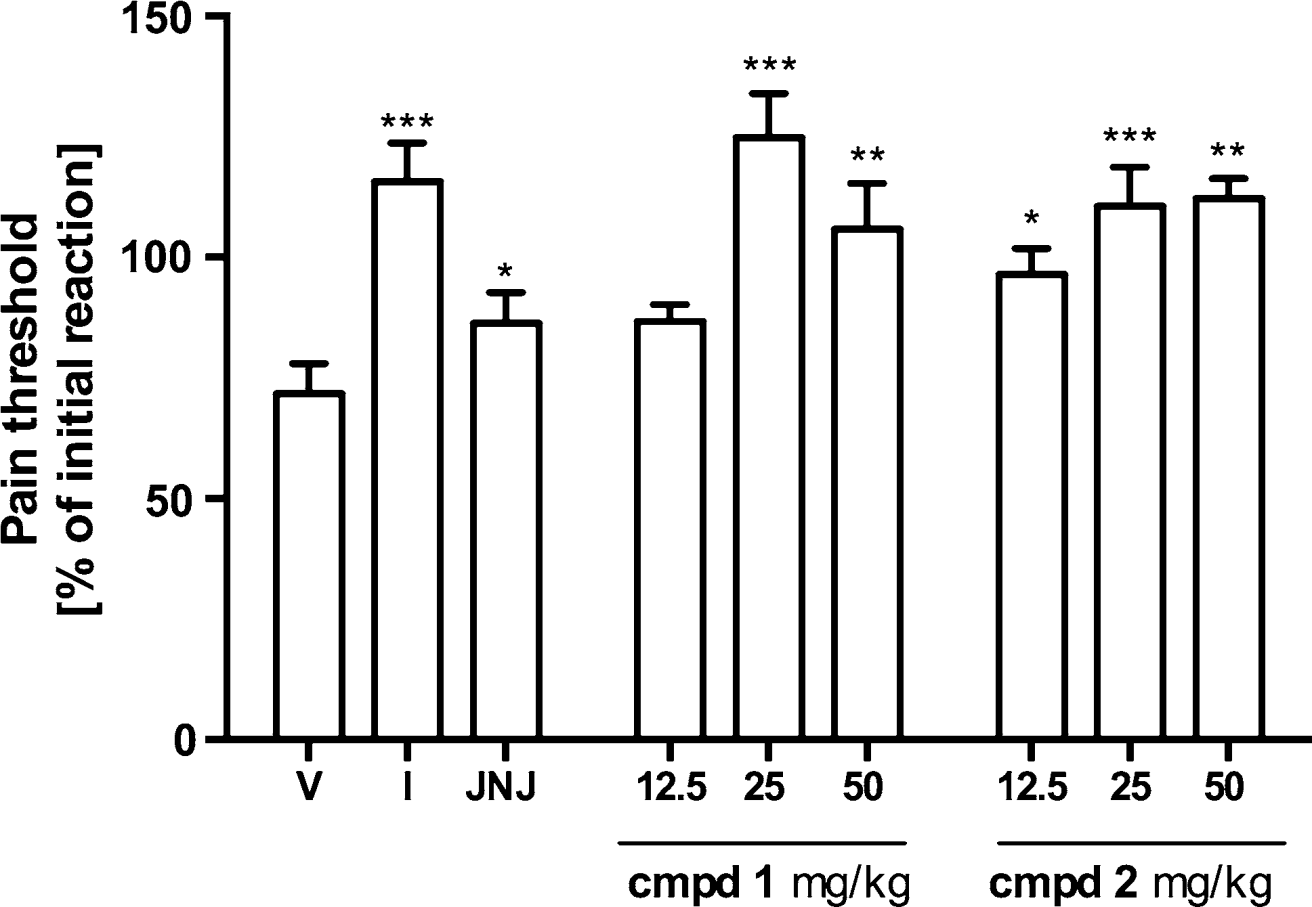
Effects of tested aryl-1,3,5-triazine derivatives, JNJ7777120 and Indomethacin on mechanical hyperalgesia developed 3 h after subplantar injection of 1 % carrageenan in rats (Randall–Selitto model). Data are expressed as mean ± S.E.M. *n* = 6–8 rats per group. The initial reaction considered as the nociceptive reaction before carrageenan administration. *V* vehicle; *I* indomethacin; Statistical significance compared to vehicle-treated animals: *p <  0.05, **p <  0.01, ***p <  0.001. Statistical analysis: one-way ANOVA followed by Dunnett’s multiple comparison test

The subplantar injection of carrageenan decreased the withdrawal threshold (mechanical hyperalgesia). The reduction peaked and reached statistical significance 3 h after the injection and was 71.74 ± 6.24 % of initial reaction. On the basis of these results, subsequent experiments evaluating mechanical hyperalgesia were carried out at this time point. Figure 3 shows that pretreatment with compound 1 resulted in the inhibition of mechanical inflammatory hyperalgesia observed as an increased withdrawal threshold. The most pronounced effect (124.87 ± 9.08 % of initial reaction) was observed at a dosage of 25 mg/kg. A statistically significant effect was also observed at a dosage of 50 mg/kg, however it was weaker (105.8 ± 9.5 %). The pretreatment with compound 2 was associated with the significant increase in pain threshold at all applied doses. The % of initial reaction were 96.4 ± 5.4, 110.6 ± 8.1, and 112.2 ± 4.2 at doses of 12.5, 25 and 50 mg/kg, respectively. The reference compounds JNJ7777123 and indomethacin were able to reduce mechanical hyperalgesia and values of initial reaction in % were for them 86.3 ± 6.5 and 115.7 ± 8.0, respectively.

**Fig. 4:**
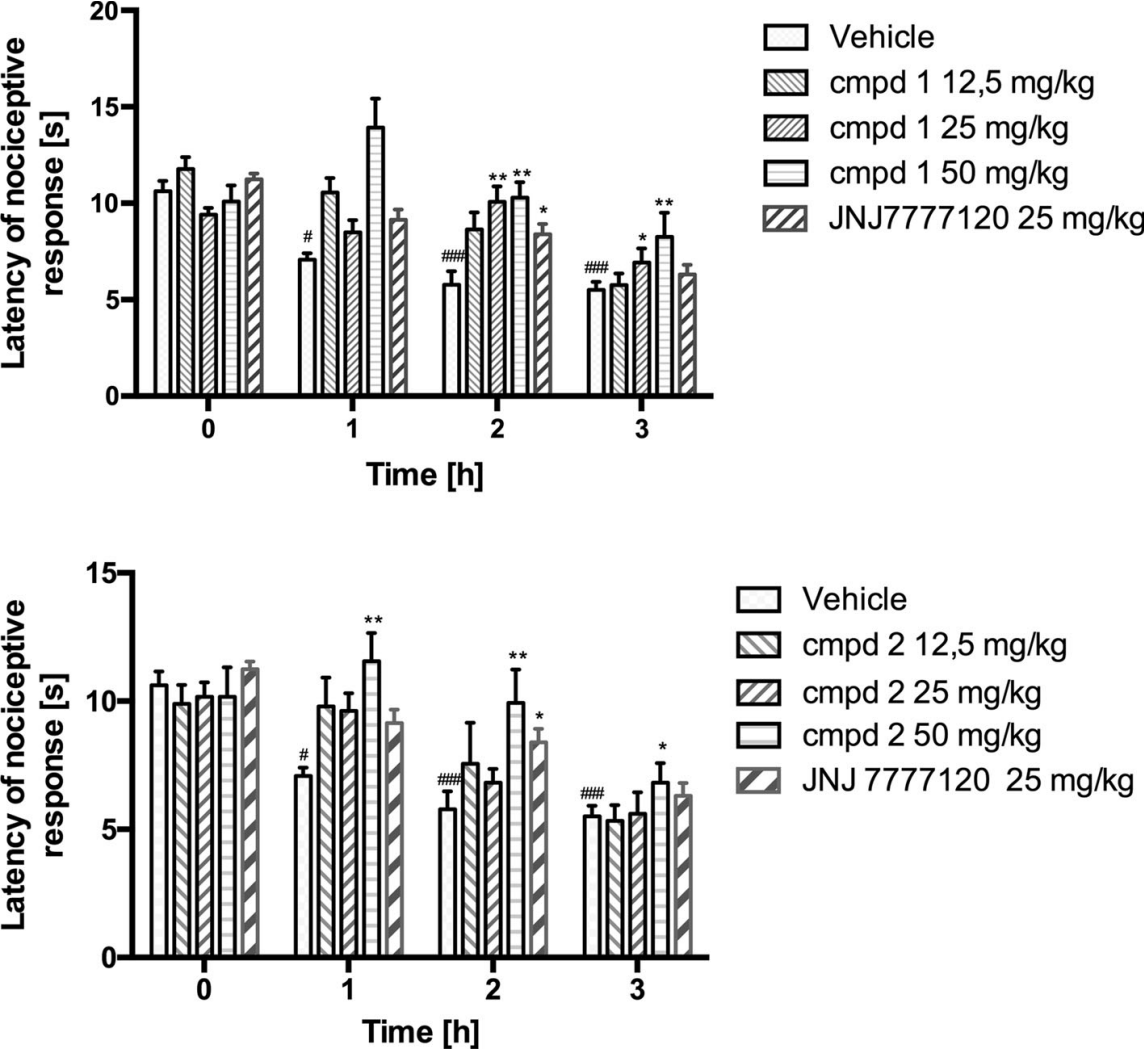
Effects of tested compounds (cmpd 1 and cmpd 2) and reference compound JNJ77120 on thermal hyperalgesia induced by subplantar injection of 1 % carrageenan in rats. Data are expressed as mean ± S.E.M. *n* = 6–8 rats per group. Statistical analysis: two-way ANOVA followed by post hoc Bonferroni test. Statistical significance compared to vehicle-treated animals: *p <  0.05, **p <  0.01, ***p <  0.001. Statistical significance in different time points of control group. ^#^ *p* <  0.05, ^###^ *p* <  0.001. Statistical analysis: ANOVA followed by Dunnett’s multiple comparison test

The subplantar injection of carrageenan induced thermal hyperalgesia observed as decreased latency of nociceptive response for radiant heat stimulation. The mean initial latencies (before carrageenan injection) were within a range of 10.62 ± 0.54 s and in the control group were significantly reduced to the values of 7.08 ± 0.32 (s), 5.78 ± 0.70 (s) and 5.50 ± 0.42 (s) 1, 2 and 3 h following carrageenan injection, respectively. As shown in Fig. 4, compound 1 was significantly active at a dosage of 25 mg/kg increasing the latency time up to 8.49 ± 0.63 (s), 10.07 ± 0.80 (s) and 6.92 ± 0.73 (s) 1, 2 and 3 h after carrageenan administration, respectively. The same values obtained after administration of compound 1 at a dosage of 50 mg/kg were 13.93 ± 1.50 (s), 10.29 ± 0.80 (s) and 8.25 ± 1.26 (s). Compound 2 showed statistically significant activity only at a dosage of 50 mg/kg, increasing the latencies up to 11.56 ± 1.10 (s), 9.93 ± 1.30 (s) and 6.82 ± 0.76 (s). The reference compound JNJ7777120 at a dosage of 25 mg/kg increased latency of nociceptive response (8.39 ± 0.53 s) in a statistically significant way only 2 h after carrageenan administration.

### Zymosan-induced peritonitis

**Fig. 5:**
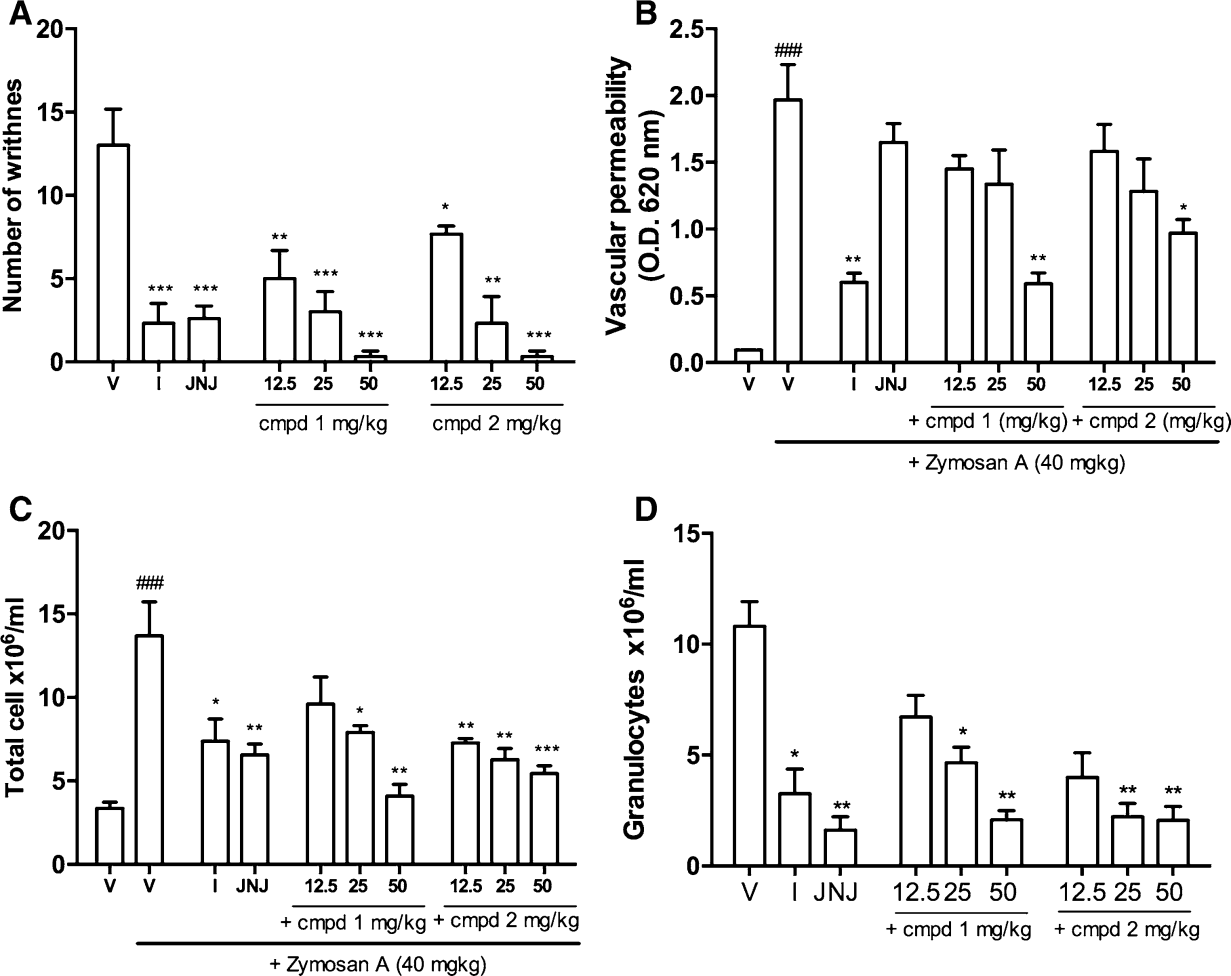
Effects of tested aryl-1,3,5-triazine derivatives and reference compounds on selected parameters of zymosan-induced peritoneal inflammation in mice. **a** The effect on the cumulative number of the pain symptoms counted during 45 min after zymosan injection. **b** The effect on vascular permeability 30 min after zymosan injection. **c** The effect on the number of total peritoneal cells 4 h after zymosan injection. **d** The effect on the number of peritoneal granulocytes 4 h after zymosan injection. Data are expressed as mean ± S.E.M. *n* = 6–8 mice per group. *V* vehicle, *I* indomethacin; Statistical significance compared to vehicle-treated animals: *p <  0.05, **p <  0.01, ***p  <  0.001. Statistical analysis: one-way ANOVA followed by Dunnett’s multiple comparison test. Statistical significance compared to Control group (animals injected only with the vehicle): ^###^ *p* < 0.001. Statistical analysis: student *t* test

The intraperitoneal injection of zymosan-induced nociceptive response observed as body writhes. The average number of them in the control group was 15.9 ± 2.1. Subcutaneous administration of the vehicle (DMSO/PBS 1:3) decreased the nociceptive response to 13.0 ± 2.2 but this effect was not statistically significant. Figure 4, panel A shows that pretreatment with test compounds as well as reference compounds resulted in a significant decrease in nociceptive response compared to the vehicle-treated animals. Compound 1 was active at all tested doses and significantly reduced the number of writhnes by 61.5, 76.9 and 97.5 % at a dosage of 12.5, 25.0 and 50.0 mg/kg, respectively. Similar effects were observed for compound 2 which decreased pain response by 41.1, 82.1 and 96.7 % at the same dosage as in the case of compound 1. In the same experiment, subcutaneous administration of a non-steroidal anti-inflammatory drug–indomethacin (10 mg/kg s.c) and reference H_4_R antagonist JNJ7777120 (25 mg/kg s.c) also elicited a statistically significant inhibition of the nociceptive response by 82.1 and 80.0 %, respectively. Moreover, 4 h after zymosan injection mice develop peritonitis, resulting in significant leukocyte accumulation in the peritoneum (Fig. 4, panel C). The lavages of peritoneal cavity of mice injected with vehicle contained 3.36 ± 0.36 × 106 cells/ml. The number of cells was several times higher after zymosan injection and reached 13.7 ± 2.0 × 106 cells/ml. Administration of investigated compounds 30 min prior to injection of zymosan resulted in the inhibition of cells accumulation in peritoneal cavity. Administration of compound 1 produced dose-dependent decrease in cell amount in peritoneal lavages, which was 9.60 ± 1.61 × 10^6^, 7.90 ± 0.41 × 10^6^, 4.10 ± 0.70 × 10^6^ cells/ml at doses 12.5, 25.0 and 50.0 mg/kg, respectively. This corresponds to 29.9, 42.3 and 70.1 % decrease in cell counts. Under the same conditions, compound 2 produced the decrease in cell amount to the values of 7.72 ± 0.27 × 10^6^, 6.28 ± 0.65 × 10^6^ and 5.45 ± 0.45 × 10^6^ cells/ml, which corresponds to 43.7, 54.2 and 60.2 % decrease in cell counts. Cell migration was also decreased by indomethacin. The effect of indomethacin was statistically significant and the number of cells was 7.44 ± 1.10 × 10^6^ cells/ml. The reference JNJ7777120 at the dose of 25 mg/kg elicited decrease in cell counts to the value of 6.55 ± 0.66 × 10^6^ cells/ml (52.2 % decrease). The analysis by flow cytometry revealed that granulocytes were the main population responsible for cell migration and accumulation. Furthermore, all of the investigated compounds had inhibitory effect on their migration (Fig. 4, panel D). There were no significant changes in the cell number of other investigated populations such as macrophages, dendritic cells or monocytes. However, some downward trends in the amount of dendritic cells were noticed. The intraperitoneal administration of zymosan-induced vascular permeability leading to the plasma protein exudation, which peaked 30 min after the injection. As it is shown in Fig. 5 (panel B), both of the investigated compounds caused significant reduction of zymosan-induced vascular permeability but only at the highest applied doses (50 mg/kg). The most profound effect was observed for compound 1, which decreased plasma exudation by 64.2 % and was similar to the effect elicited by indomethacin, which decreased plasma exudation by 63.6 % at a dosage of 10 mg/kg. Compounds 1 and 2 as well as JNJ7777120 were not active when given at a dosage of 12.5 or 25 mg/kg.

### Influence on spontaneous locomotor activity

The mean number of light-beam crossings in the vehicle-treated animals was 1.46 ± 0.2 × 10^3^ measured during the whole 30 min-long period of observation. The value was not significantly altered in the animals treated with test compounds at the dose of 50 mg/kg.

### Influence on motor coordination in the rotarod test

In the rotarod test the vehicle-treated mice did not demonstrate any signs of impaired motor coordination. The time spent on the rotarod apparatus was 60 s for each control mouse. The same effect was observed for the compound-treated mice. Neither compound 1, nor compound 2, impaired motor coordination of mice in the rotarod test at administrated dose (50 mg/kg) and any tested speed.

### Influence on LPS-stimulated RAW 264.7 cells

**Fig. 6:**
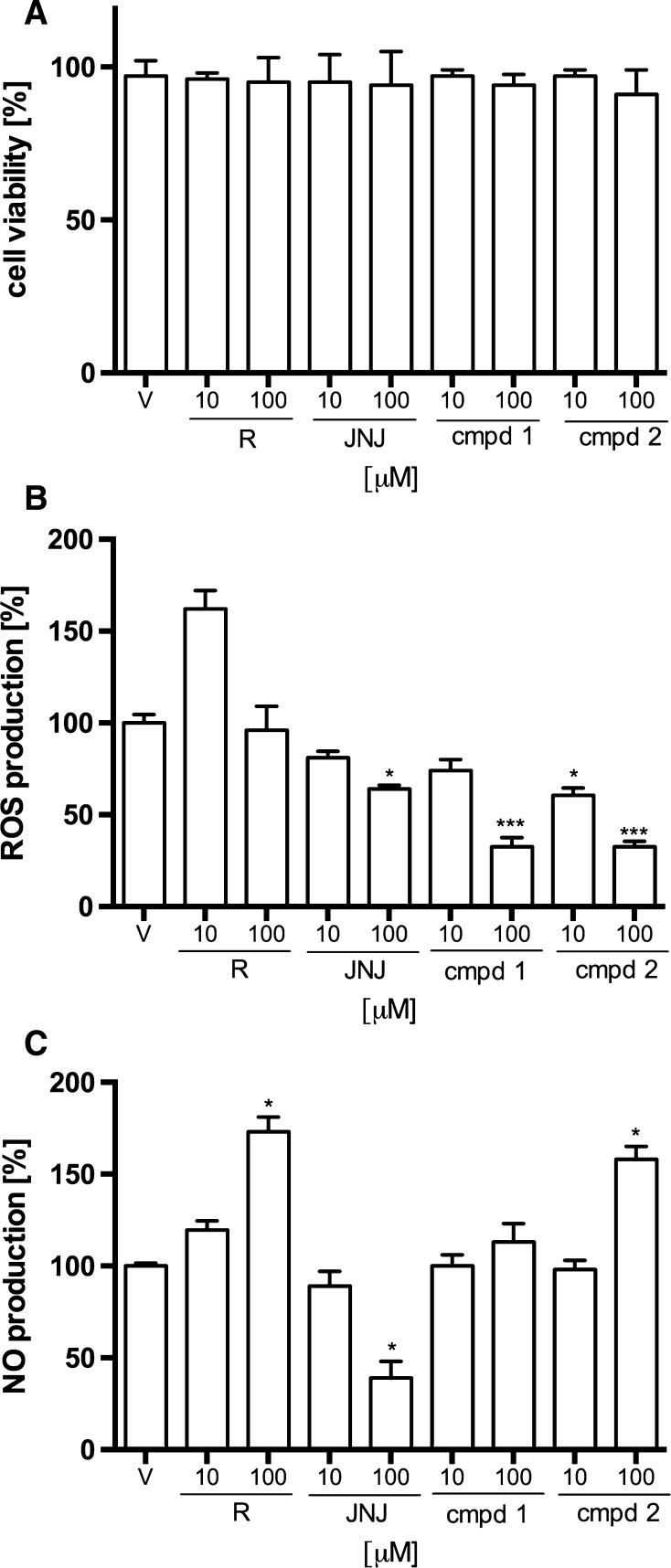
Effect of tested aryl-1,3,5-triazine derivatives and reference compounds (*R* Rolipram, *JNJ* JNJ7777120) on selected parameters of LPS-stimulated RAW 264.7 cells. **a** Effect of compounds on cell viability **b** Effect of the compounds on LPS-induced production of reactive oxygen species (ROS). **c** Effect of the compounds on LPS-induced synthesis of nitric oxide (NO). Data are expressed as mean ± SEM of at least 2 independent experiments, which were run in triplicates. Statistical significance compared to vehicle-treated animals: *p <  0.05, **p <  0.01, ***p < 0.001. Statistical analysis: one-way ANOVA followed by Dunnett’s multiple comparison test

The potential cytotoxicity of the investigated aryl-1,3,5-triazine derivatives was evaluated after incubating cells for 24 h in the absence or presence of LPS. The cytotoxic effects of cmpd 1 and cmpd 2 as well as reference compounds have been presented in Fig. 6a. The results show that neither test compounds nor reference compounds exhibited any significant toxicity in RAW 264.7 cells at the concentration of 10 and 100 μM. Thus, further effects were not attributable to cytotoxic effects of the studied compounds.

The levels of ROS and NO production in LPS-stimulated RAW 264.7 cells were determined. As shown in Fig. 6b the incubation of cells with JNJ7777120 or investigated compounds resulted in decreased production of ROS compared to the control (LPS-stimulated cells incubated with vehicle). The most pronounced and statistically significant effect was observed at a concentration of 100 μM. The ROS production decreased to the level of 64.0 ± 2.1 % of control, 32.5 ± 5.0 % of control and 32.5 ± 3.0 % of control, respectively, for JNJ7777120, compound 1 and compound 2. Moreover, compound 2 inhibited ROS production in a statistically significant way at a concentration of 10 μM (60.5 ± 4.0 % of control). JNJ7777120 and compound 1 at a concentration of 10 μM decreased ROS production (81.0 ± 3.5 and 74.0 ± 6.0 % of control) but the effect was not statistically significant. The incubation of cells with Rolipram was not associated with decreased production of ROS. Figure 6c shows that the only compound, which decreased the NO production in LPS-stimulated RAW 264.7 cells was JNJ7777120. This reference compound decreased NO production to 39.0 ± 9.0 % of control at a concentration of 100 μM, whereas the NO production was reduced to 89.5 ± 8.0 % of control at a concentration of 10 μM. The compound 1 had no significant influence on NO production, whereas compound 2 increased NO production to the level of 158 ± 7.0 % of control at a concentration of 100 μM. This effect was similar to that obtained for Rolipram, which increased NO production to the level of 173 ± 8.0 % of control at the same concentration.

**Fig. 7:**
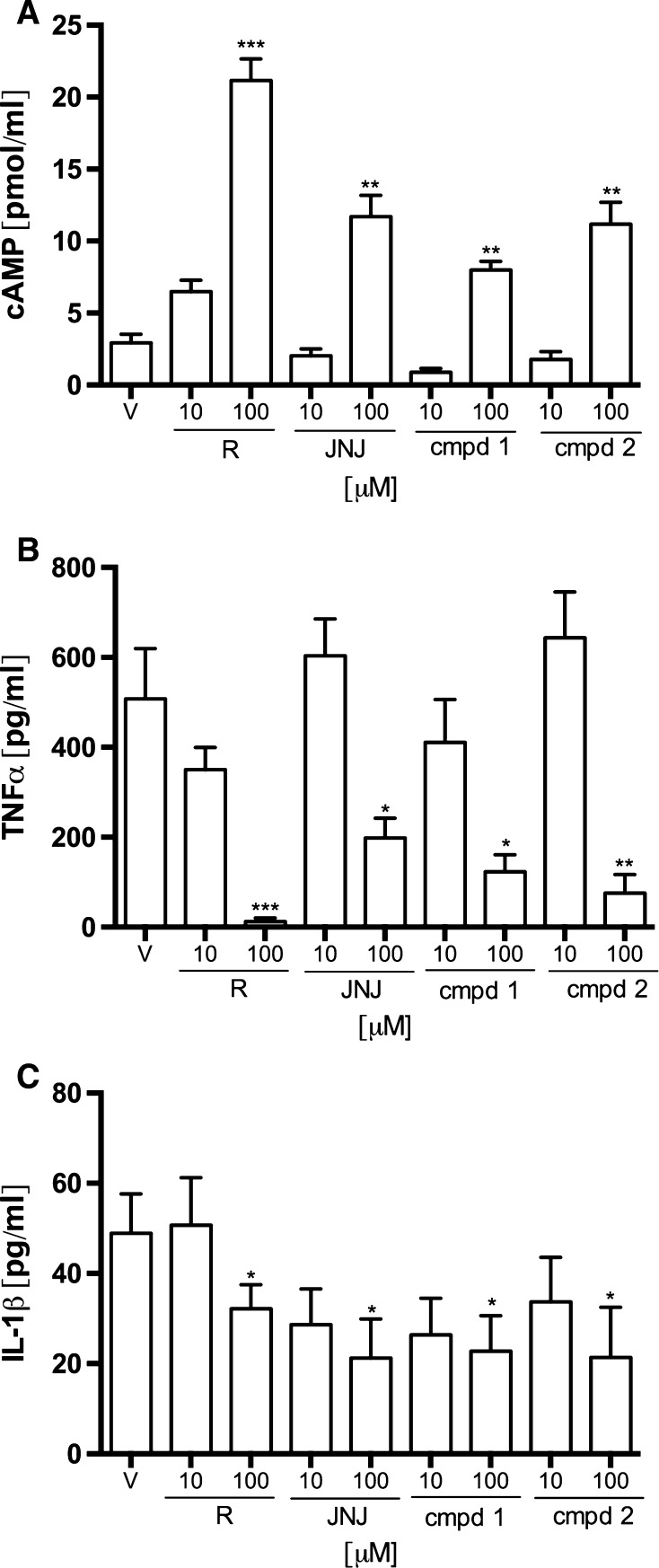
Effect of tested aryl-1,3,5-triazine derivatives and reference compounds (*R* Rolipram, *JNJ* JNJ7777120) on cAMP and cytokine production in LPS-stimulated RAW 264.7 cells. **a** Effect of compounds on the concentration of cAMP in the lysate of RAW 264.7 cells. **b** Effect of the compounds on LPS-induced release of TNFα. **c** Effect of the compounds on LPS-induced release of IL-1β. Data are expressed as mean ± SEM of at least two independent experiments, which were run in triplicates. Statistical significance compared to vehicle-treated animals: *p <  0.05, **p <  0.01, ***p < 0.001. Statistical analysis: one-way ANOVA followed by Dunnett’s multiple comparison test

As shown in the Fig. 7a, the concentration of cAMP, measured in the cell lysate, was increased in the presence of all investigated compounds at the concentration of 100 μM. The concentration of cAMP in the control group was 2.91 ± 0.61 pmol/ml and increased to the value of 21.16 ± 1.50 pmol/ml (727 % increase) after incubation with Rolipram at a concentration of 100 μM. Incubation with the same compound at a concentration of 10 μM resulted in the increase of cAMP concentration to the value of 6.48 ± 0.81 pmol/ml (223 % increase). At concentration of 10 μM, both reference antagonist of H_4_R (JNJ7777120) and studied compounds did not significantly altered cAMP concentration. Nevertheless, at the concentration of 100 μM, these compounds increased cAMP to the values of 11.69 ± 1.49 pmol/ml (402 % increase), 7.98 ± 0.61 pmol/ml (274 % increase) and 11.17 ± 1.52 pmol/ml (384 % increase), respectively, for JNJ7777120, compound 1 and compound 2.

TNF-α and IL-1β concentrations in the culture supernatants of RAW 264.7 cells were measured by ELISA method. RAW 264.7 cells treated with LPS released significant amounts of the examined cytokines. The concentration of TNF-α increased up to 508 ± 112 pg/ml after LPS stimulation, whereas the concentration of IL-1β increased up to 48.9 ± 8.7 pg/ml from the undetectable level in the cell culture without LPS. The concentrations of TNF-α and IL-1β in the supernatant of cells pretreated with 100 μM of compound 1, compound 2, Rolipram and JNJ7777120 were significantly decreased compared to the vehicle-treated group. The incubation with Rolipram resulted in the reduction of TNF-α to the concentration of 11.6 ± 7.9 pg/ml. JNJ7777120 decreased the TNF-α concentration to the value of 198 ± 44 pg/ml and compound 1 and compound 2 to the values of 122 ± 44 and 76 ± 41 pg/ml. The investigated compounds, at the concentration of 10 μM, did not have a statistically significant effect compared to the control group. Although the most potent in inhibiting TNF-α release, Rolipram showed slightly lower activity than the investigated compounds concerning the inhibition of IL-1β release. The concentration of this cytokine was 32.2 ± 5.3 pg/ml for Rolipram and ranged from 21.2 ± 8.7 pg/ml for JNJ7777120 to 22.8 ± 7.9 pg/ml for compound 1.

### Histamine H_1_ receptor functional assay

**Fig. 8:**
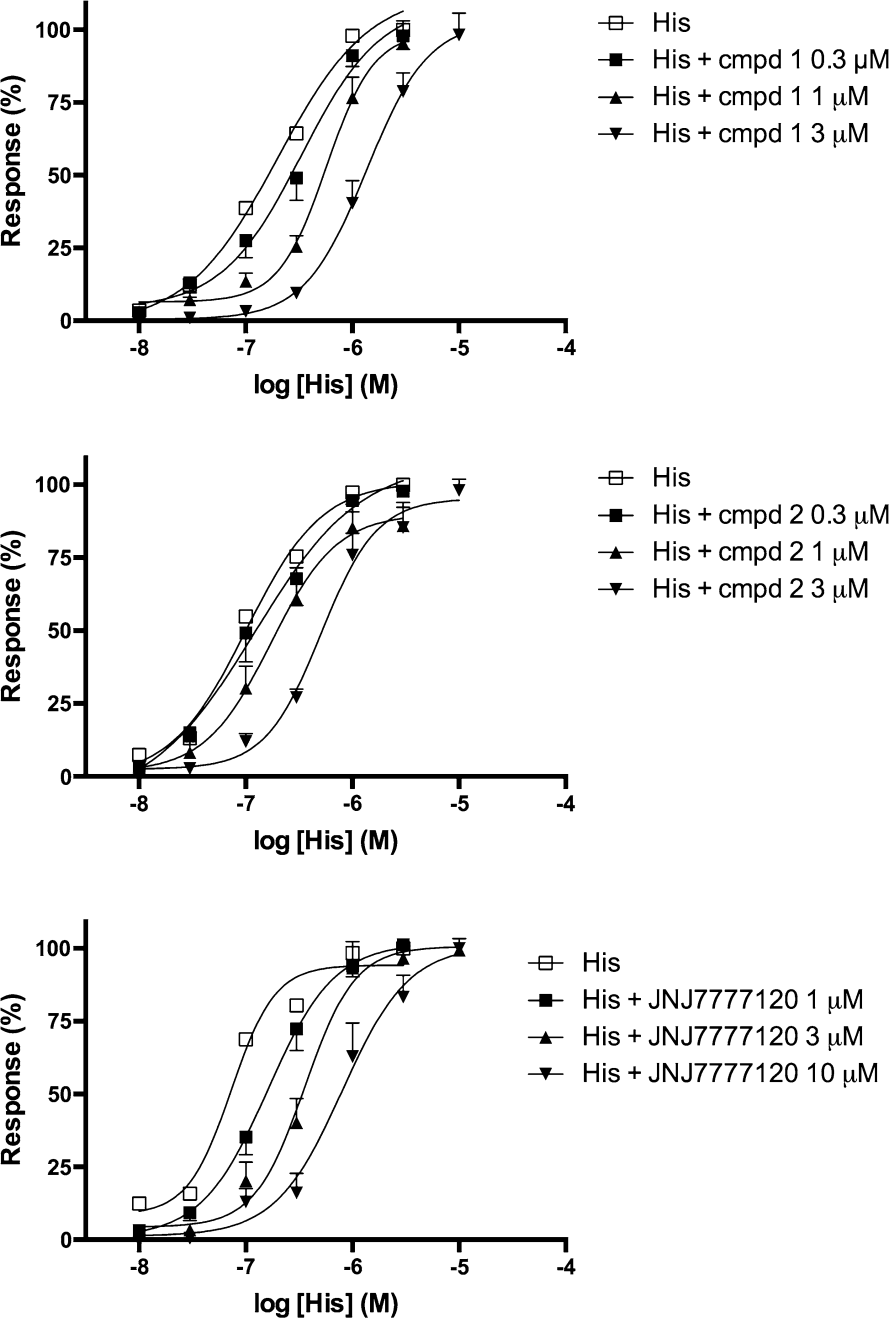
Effect of tested compounds (1 and 2) and antazoline on H_1_ histamine receptors. Concentration-response curves to histamine (His) in the absence or presence of increasing concentrations of compounds. Results are expressed as percentage of the maximal response to histamine in the corresponding concentration-response curve. Each* point* represents the mean ± SEM (*n* = 6–8)

The effect of compounds 1 and 2 on the histamine-induced contractions was measured in guinea-pig ileum. The tested compounds alone had no ability to induce contractions of ileum. The contractile response to agonist (histamine) was inhibited by the tested compounds in a concentration-dependent manner without affecting the maximum response (Fig. 8). The pA_2_ values were obtained with a Schild regression slope not significantly different from unity, indicating a competitive interaction of the compounds with the histamine H_1_ receptors present in this tissue. However, the effect was rather weak. Both compounds 1 and 2 revealed similar antagonistic activities, which were also similar to the activity of JNJ7777120. For compound 1 the pA_2_ value was 6.320 ± 0.03 (*s* = 0.94 ± 0.03), for compound 2 it was 5.953 ± 0.07 (*s* = 1.29 ± 0.15), and for JNJ7777120 it was 6.111 ± 0.07 (*s* = 0.92 ± 0.05). Under the same experimental condition, antazoline revealed significantly stronger antagonistic potency: the pA_2_ value was 7.142 ± 0.11 (*s* = 1.11 ± 0.08). Detailed results are presented in Table 1.

**Table 1:** Functional affinities of test compounds and reference compounds for histamine H_1_ receptors expressed in in guinea-pig ileum

Compound	pA_2_ ± SEM	Slope ± SEM
Cmpd 1	6.320 ± 0.03	0.94 ± 0.03
Cmpd 2	5.953 ± 0.07	1.29 ± 0.15
Antazoline	7.142 ± 0.11	1.11 ± 0.08
JNJ7777120	6.111 ± 0.07	0.92 ± 0.05

Antagonist potency expressed as pA_2_ ± SEM or pK_B_ ± SEM

### The phosphodiesterase 4B1 (PDE 4B1) assay

The inhibitory activity of compound 1 and compound 2 toward PDE 4B1 enzyme was measured using the bioluminescent detection system, based on the activity of PDE which utilized cAMP as its preferential second messenger. Neither investigated compounds nor reference antagonist of H_4_R JNJ7777120 had significant inhibitory potencies for PDE 4B1 in applied concentrations, whereas reference compound Rolipram decreased the PDE activity by 95 and 98 % at a concentration of 50 and 100 μM, respectively. Moreover, irsogladine, selective PDE 4 inhibitor chemically close to the structure of compound 1 and 2, inhibited PDE activity in 20 and 31 % at a concentration of 50 and 100 μM, respectively. All the results are presented in Table 2.

**Table 2:** Inhibitory effect of investigated and reference compounds for PDE 4B1 (%)

Compound	Concentration (μM)	PDE 4B1 inhibition (%) ± SEM
Cmpd 1	50	7 ± 1.0
	100	9 ± 1.0
Cmpd 2	50	2 ± 0.0
	100	6 ± 1.0
JNJ7777120	50	0 ± 0.0
	100	1 ± 0.5
Irsogladine	50	20 ± 2.5
	100	31 ± 0.5
Rolipram	50	95 ± 0.0
	100	98 ± 0.0

Percentage of PDE inhibition was calculated in relation to the vehicle control (DMSO)

## Discussion

We found that new H_4_ receptor antagonists attenuated inflammatory and nociceptive response in two in vivo models of inflammation. Both compounds (cAMP dependently) inhibited inflammatory mediators release and ROS production in RAW 264.7 cells.

Inflammation and inflammatory pain are very complex processes associated with the release of numerous inflammatory mediators. Histamine is one of the most important autacoid engaged in the formation of inflammatory response. This mediator, acting also as neurotransmitter, exerts its function through four different types of GPCRs: H_1_R, H_2_R, H_3_R and H_4_R [13]. The identification of H_4_Rs, the newest receptors in the group, and their fairly selective expression on cells involved in inflammatory and immune responses suggested their important role in inflammation [3, 28]. The subsequent discovery of the potent and selective H_4_R antagonist-JNJ7777120 [29]-enabled studies on physiological and the pathophysiological functions of H_4_R and provided the first evidence that H_4_R blockade could result in anti-inflammatory effect [30]. In recent years, scientists reported that H_4_R antagonists are effective in models of asthma, dermatitis, arthritis, pain, pruritus and colitis [17–20, 31–33].

Despite numerous studies on histamine H_4_R, the differences between species in response to specific ligands remain ambiguous. Most current data indicate that JNJ7777120 is H_4_R antagonist. However, researchers proved that in some species and transfected cell models, the compound acted as H_4_R agonist [8–10]. Moreover, it exerted functional selectivity, i.e., β-arrestin activation [8–10]. Thus, in our opinion, research into H_4_R-dependent pharmacological effects are valuable.

Our present research investigated pharmacological activity of the two previously synthesized aryl-1,3,5-triazine derivatives (compound 1 and compound 2) with affinity for histamine H_4_R. Previous studies demonstrated that both compounds showed submicromolar affinity and antagonist potency at hH_4_R (compound 1, Ki-203 nM, IC_50_-512 nM; compound 2, Ki-524 nM, IC_50_-1630 nM) as well as good selectivity over hH_3_R. Additionally, preliminary pharmacological experiments demonstrated their anti-inflammatory properties (carrageenan-induced paw edema in mice) and lack of antiproliferative effect (in HEK-293 and IMR-32 cell lines) [21].

To evaluate anti-inflammatory and anti-nociceptive properties of compounds, we used two in vivo models of inflammation (carrageenan-induced model of inflammation and zymosan-induced peritonitis). We intentionally performed the experiments on mice and rats, to demonstrate that the compounds show effects regardless of the species and the related variability in expression and activity of the histamine H_4_R.

In the carrageenan-induced model of inflammation both test compounds reduced edema in all time points. Since Coruzzi and colleagues reported that H_4_R antagonists were effective only at the acute inflammatory response in this model of inflammation [19], we measured the activity of the compounds during the first 3 h of the test. In this period, the compounds showed greater anti-inflammatory properties than the reference compound-JNJ777120 (compared at the same doses). Nevertheless, the investigated compounds at the dose of 50 mg/kg were not as potent as indomethacin at the dose of 10 mg/kg. The obtained results prove that the blockade of H_4_R function results in an anti-edematous effect.

We confirmed the anti-inflammatory activity of investigated aryl-1,3,5-triazine derivatives in the model of zymosan-induced peritonitis. Administration of test compounds resulted in attenuated vascular permeability and decreased intraperitoneal influx of inflammatory cells. The pretreatment with the highest doses of compounds (50 mg/kg) reduced vascular permeability, whereas lower doses and JNJ7777120 had no significant effect. Given the lower affinity of triazines for H_4_R compared with JNJ7777120, we suggest that decreased vascular permeability was not associated with the direct influence on H_4_R. We think that it resulted from other mechanisms e.g., decreased release of inflammatory mediators such as leukotrienes or cytokines, which regulate vascular permeability during inflammation [23]. The inhibitory effect of H_4_R antagonist on cell migration (especially neutrophils) in zymosan-induced inflammation is well known [30]. However, other cells also express H_4_R i.e., hematopoietic progenitor cells [14] mast cells, eosinophils [5], Th_2_ lymphocytes [31], monocytes, dendritic cells [34–36], Natural killer cells [15], monocytes, macrophages and some other cell types [13]. Thus, we assessed the amount of various cell populations involved in inflammatory response in peritoneal lavage. In fact, we showed significantly decreased granulocytes migration. However, we observed a tendency in decreasing the number of NK cells and dendritic cells (data not published). We think that evaluating cell number in peritoneal cavity in additional time points would clarify the role of H_4_R antagonist in other cell type migration.

The test compounds inhibited both, thermal and mechanical hyperalgesia induced by carrageenan injection. As Figs. 3 and 4 present their effect was stronger and lasted longer than that of JNJ7777120. This is particularly visible in case of compound 1. The compounds also reduced body writhnes in the model of zymosan-induced peritonitis (Fig. 5 panel A). In both models, nociceptive reactions are not related to the direct stimulation of nociceptors, but rather result from the secondary release of the inflammatory mediators such as prostanoids or cytokines from immunocompetent cells [23, 37]. The majority of these cells shows expression of H_4_R [2]. Although the contribution of H_4_R in the mechanism of pain still remains controversial, studies confirm that H_4_R antagonists elicit analgesic effects in inflammatory and neuropathic pain models [19, 20]. In contrast, recently published papers revealed that H_4_R stimulation exhibited pain-reducing effects [38–40]. Taken together, all these data suggest that H_4_R antagonists might possess anti-hyperalgesic properties that are secondary to decreased release of inflammatory mediators, whereas activation of neuronal H_4_Rs, especially those localized on sensory dorsal root ganglion neurons, might result in antinociceptive effects in the absence of inflammation [39].

Thus, we concluded that the anti-inflammatory and anti-hyperalgesic activity of the investigated compounds resulted from their secondary and H_4_R-dependent inhibitory influence on the release of inflammatory mediators. To confirm this hypothesis, we assessed the influence of test compounds on LPS-stimulated RAW 264.7 macrophages. The activation of macrophage TLR-4 (Toll-like Receptor) by LPS induces the expression of the histamine-generating enzyme l-histidine decarboxylase and subsequent histamine synthesis [16]. Histamine, released form macrophages, activates H_4_R leading to a decreased level of intracellular cAMP among others [13].

Both aryl-1,3,5-triazine derivatives incubated with RAW 264.7 increased intracellular cAMP concentration and significantly decreased TNFα and IL-1β release. Moreover, test compounds decreased reactive oxygen species (ROS) level stronger than JNJ7777120. In the same conditions, JNJ7777120 attenuated NO production, while compound 1 had no effect and compound 2 increased NO formation. Compound 2 increased NO formation similarly to rolipram.

The impact of the tested compounds on NO synthesis was not in line with the result obtained for JNJ7777120, which indicates that they may alter some other pathways involved in NO synthesis. NO is generated by inducible NO synthase (iNOS) in macrophages following exposure to cytokines or microbial products, such as LPS [41]. Furthermore, a vast amount of NO can cause tissue damage and contribute to the development of a wide spectrum of inflammatory diseases.

Under inflammatory conditions ROS open inter-endothelial junctions and promote the migration of inflammatory cells across the endothelium of postcapillary venules. Therefore, we believe that ROS formation inhibition is an important mechanism of anti-inflammatory activity of the investigated aryl-1,3,5-triazine derivatives. Additionally, ROS play role in edema formation by inducing the paracellular permeability, which is the major route of vascular leakage observed in a variety of inflammatory states and is associated with the extravasation of protein-rich fluid from the luminal to abluminal side of the endothelium [42]. Decreased level of ROS might also contribute to the antinociceptive effect of the test compounds. ROS evoke nociceptive response in neurogenic inflammation through different targets including TRPA1 receptors [24].

As described above, the tested compounds increased cAMP level, which was similar to JNJ7777120. The increased concentration of cAMP in LPS-stimulated RAW 264.7 observed after their incubation with JNJ7777120 or the test compounds might result from H_4_R blockade. The same mechanism might contribute to the reduced release of pro-inflammatory cytokines such as IL-1β and TNFα [2, 31, 43, 44]. This effect, in turn, might be involved in the antinociceptive activity of the tested compounds [45–48].

Altogether, our results suggest that the influence of JNJ7777120 on NO production is cAMP-independent and may result from the activation of β-arrestin-dependent pathway. Since, in contrast to JNJ7777120, the compounds increased NO production, we hypothesize that they did not activate β-arrestin-dependent pathway. However, the effect of cellular cAMP on NO release is still not confirmed, since elevation of cAMP level can either stimulate or inhibit NO formation [41, 49]. The increased level of NO may be also considered as secondary effect of decreased level of ROS, since ROS such as superoxide can rapidly combine with NO to form reactive nitrogen species (RNS) [42]. The tested triazines are more potent than JNJ7777120 in reducing the ROS formation and consequently they may prevent the ROS-dependent inactivation of NO.

Neurotoxicity or potent sedative effect of the new compounds can limit their future utility, and what is more, result in ambiguous or incorrect interpretation of the results of in vivo tests. Our experiments investigating the influence of the test compounds on spontaneous locomotor activity and their influence on motor coordination in rotarod test revealed that all in vivo effects of compounds were observed at the doses that did not cause neurologic deficits and did not significantly reduce spontaneous locomotor activity.

Although both studied compounds possess significantly lower affinity for H_4_R than JNJ7777120, their activity in attenuating inflammatory and nociceptive response was comparable to the reference compound. We propose two main explanations of this phenomenon. The first is species difference in the H_4_R structure and function. The affinity of the test compounds for H_4_R was assessed in human receptors, whereas all experiments were carried out on mice and rats. In our opinion, it is unlikely that the affinity for mice receptors would be much higher than for human receptors. On the other hand, the species differences might be partially responsible for this effect. Some histamine H_4_R ligands act as inverse agonists at the human H_4_R, which is constitutively active, whereas as neutral antagonists at the constitutively inactive mouse and rat H_4_R [11]. Moreover, the test compounds and JNJ 7777120 may vary in their impact on β-arrestin-dependent intracellular pathway. JNJ 7777120 appears to be a partial agonist in β-arrestin recruitment [9]. In case of test compounds, at this stage of research it is difficult to assess their character of interaction with H_4_Rs on the molecular level.

The second explanation is that some additional mechanisms are involved in the final effect of the investigated compounds. The experiments on isolated ileum suggested that we cannot definitely exclude the potential involvement of histamine H_1_R, since the investigated compounds produced a slight shift of the histamine concentration-response curve. The obtained pA_2_ values (see Table 1) indicate that the affinity of compound 1 and compound 2 for H_1_R was rather low. Nevertheless, even weak antagonism at H_1_R might decrease vascular permeability [22]. This might be particularly important when high doses are administered.

We showed that the influence on cAMP level in RAW 264.7 cells was similar for the test compounds and JNJ7777120. Taking into account the lower affinity of the compounds for H_4_R, we suggest that phosphodiesterase inhibition could be involved in this effect. The fact that the chemical structure of the test compounds is similar to the structure of Irsogladine—the PDE inhibitor—supports this hypothesis [50, 51]. Phosphodiesterase inhibition could be one of the pharmacological mechanisms of action of 1,3,5-triazine derivatives. PDE inhibitors elicit similar effects to those observed for the investigated compounds. Non-selective inhibition of PDE could explain the impact of these compounds on NO synthesis, since it might increase cGMP level and subsequently activate an important regulator of the activity of NO synthase (NOS)-sGC (soluble guanylyl cyclase). Several studies showed that either PDE4-specific inhibitors such as rolipram [52–54] or non-selective PDE inhibitors such as pentoxifylline or theophylline [55–58] were effective in attenuating pain and inflammation. Moreover, the antinociceptive activity of non-selective [56] as well as selective [54] PDE inhibitors was associated with the decreased release of cytokines such as IL-1β and TNFα. It is widely accepted that this effect is due to the increase of intracellular concentration of cAMP as a result of PDE inhibition in inflammatory and immunocompetent cells [59]. To confirm or rule out the involvement of PDE inhibition in a mechanism of action of the investigated compounds, we investigated the inhibitory potential of studied compounds for the PDE 4B1 enzyme. PDE 4B1 is the phosphodiesterase isozyme, which is expressed in inflammatory cells, such as macrophages [54]. The obtained results allow to definitely exclude the involvement of PDE inhibition from the pharmacological activity of the investigated aryl-1,3,5-triazines. Thus, we claim that anti-inflammatory and analgesic activities of these compounds are primarily H_4_R dependent and the differences between them and JNJ7777123 are due to the different interaction with H_4_R on the molecular level.

In conclusion, we demonstrated that two new H_4_R antagonists attenuated inflammatory and nociceptive response in in vivo models of inflammation. Both compounds (cAMP dependently) inhibited inflammatory mediators release in RAW264.7 cells and ROS production. Although our results provided new insight into the pharmacological profile of H_4_R ligands, some questions remain open, which encourages further studies.
